# Situating Educational Psychology Practice. Exploring the Call for a ‘Practice Turn’ in Contemporary Danish Educational Psychology Practice

**DOI:** 10.1007/s12124-025-09915-6

**Published:** 2025-06-04

**Authors:** Sarah Kirkegaard Jensen, Thomas Szulevicz

**Affiliations:** Department of Communication and Psychology, Aalborg University, Aalborg, Denmark

**Keywords:** Educational psychology, Educational psychology practice, Institutionalized education, Practice turn, Situated theory, History, Normativity

## Abstract

In recent years, Denmark has witnessed issues related to the mental health and well-being of children and adolescents, marked by rising psychiatric diagnoses, school absenteeism, and increasing segregation in educational settings. This development has intensified the demand for Educational Psychology (EP) practice services but also started a public and professional debate about the role and effectiveness of EP practice. Critics argue that EP practice is too detached from pedagogical realities, focusing excessively on documenting individual difficulties rather than supporting educational practices. Thus, educational psychologists are requested to make a turn towards practice. Through a situated psychological lens, this article explores the desired practice turn of EP practice: what kind of practice is presented in the current educational and political discussion and what is the role of educational psychology and educational psychology practice in this raised critique of a ‘practice-distant’ EP practice? Through a situated psychological lens, the article argues that understanding the field of EP practice, including the role of educational psychologists in contemporary educational practices, requires acknowledging its historical and contextual situatedness inside the practice of institutionalized education. In addition, the article argues for a fundamental theoretical and normative discussion about the type of practices educational psychologists should support.

[T]hinking isolated from practice is a purely scholastic question

(Marx, 2. Theses on Feuerbach, cited in Bernstein, 1971, p. 11).

## The Problem of ‘Practice’ in Contemporary Danish Educational Psychology Practice

In recent years in Denmark, a steady stream of reports and studies have been published documenting the continuous deterioration of mental health and well-being of children and adolescents. Increasing numbers of children and adolescents are receiving psychiatric diagnoses (Kleding, 2023), and a growing proportion of school students exhibit concerning levels of school absenteeism (Knage & Kousholt, 2023). Additionally, expenses for special educational measures are skyrocketing in many municipalities, and the degree of segregation is increasing (Thomas & Loxley, 2022; VIVE 2022). This development has led to more referrals to municipal Educational Psychology (EP) practice offices and psychiatric services (EVA, 2023), placing increased pressure on daycare centers, schools, and other educational institutions where children and adolescents spend their daily lives, to handle, support, and resolve these problems.

In the wake of this development, the role, purpose, and function of EP practice have once again become subjects of public debate. Critics argue that EP practice is too far removed from pedagogical practice and spends too much time describing and documenting children’s individual difficulties rather than supporting pedagogical work in daycare centers and schools (Nørmark & Jensen, 2018). This debate is also reflected in the research literature on the subject, and quite interestingly, there is internal support within EP practice circles for parts of the criticism. Notably, more than 1,000 psychologists – most of whom worked in EP practice – expressed their concerns about the everyday frameworks that children are part of in daycare and schools in an open letter published in the Danish newspaper *Politiken* in the fall of 2022 (Mølgaard & Jensen, 2022). The psychologists’ letter stated their difficulties in supporting children’s wellbeing through preventive work, as most of their working time is spent making written assessments of individual children in various challenging situations. Consequently, there is a strong push for an EP practice system with more ‘practice-oriented counseling’ these years in Denmark. This need is being promoted both in educational policy by, for example, DLF,^1^ BUPL,^2^ and KL,^3^ as well as by several EP practice units themselves. It is also seen in both Danish and international research literature on EP practice (Borring, 2023; Caspersen et al., 2023; EVA, 2023, etc.), and Borring (2023) even refers to this promoted need as a ‘practice turn’ of EP practice.

Two issues, in particular, seem to underscore this requested need for a practice turn. Firstly, many EP practice offices are currently experiencing more referrals and increasing demand for individual assessments (EVA, 2023). This development means that psychologists in EP practice spend a larger proportion of their total working time writing statutory Pedagogical Psychological Assessments (PPAs). In both national and international research literature on PPAs, it is pointed out that preparing a PPA is very time-consuming and typically involves spending many resources on gathering information and describing and documenting the referred child’s conditions and challenges (e.g., Umaña et al., 2020). Despite the time-consuming nature of these reports, research shows that the professional recommendations in the reports are only to a lesser extent translated into concrete pedagogical practice (Lindelauf, 2022; Lindelauf et al., 2018, Mastoras et al. 2011; Rahill, 2018; Szulevicz & Arnfred, 2022, 2023; Vaags & Uthus, 2025). Overall, the increased number of referrals to EP practice means that employees spend more time documenting difficulties than addressing them in practice in daycare centers and schools.

Secondly, there is frequent criticism of EP practice employees’ so-called ‘practice-oriented competencies’ (Højholt & Szulevicz, 2013; Kousholt & Morin, 2023; Szulevicz & Tanggaard, 2015). It is pointed out, among other things, that EP practice lacks insight into and knowledge of fundamental pedagogical and didactic conditions, resulting in EP practice counseling that is too abstract and disconnected from the everyday practices in school and daycare. Some of the criticism is expressed for example in EVA’s recently published report on EP practice:

What is said to be missing is a connection between the psychological theoretical knowledge and a practice-oriented understanding of the school context and the competence to translate theoretical insights into this practice (EVA, 2023, p. 61).

Hence, the ‘psychological theoretical’ knowledge that many EP psychologists possess is criticized for being too difficult to connect with the ‘practice-oriented’ understanding of local, pedagogical everyday life in daycare and schools. This criticism has led to a heated educational policy discussion about which professional skills are most relevant in EP practice to achieve the desired turn towards practice.

In this article we explore another, yet related, interest: what kind of ‘practice’ is presented in the current educational and political discussion outlined above? Additionally, what is the role of educational psychology and educational psychology practice in this raised critique of a ‘practice-distant’ EP practice?

## The Situating Purpose of the Article

The aim of this article is to explore how we can understand this requested turn towards practice of contemporary Danish EP practice and what it could potentially entail. We have three reasons for this exploration:

*First*, conceptualizations of ‘practice’ and a ‘practice turn’ in and around contemporary EP practice, as outlined in the political discussion above, are often disconnected from an understanding of EP practice as deeply embedded in the historical practice of institutionalized education. However, the ‘practice’ that educational psychologists are said to be detached from, is not merely a neutral setting or location where education ‘takes place’; it is a historically and socially produced normative practice (Bernstein, 1971; Lave & Packer, 2008). This disconnection poses significant risks: A turn towards practice for EP practice predominantly comes to mean being ‘in’ practice (e.g. sitting in the classroom) as opposed to, for example, examining and assessing children ‘outside’ of their everyday practice. Here, practice is reduced to a physically confined place, a ‘container’ around a schoolchild or a school class, which one can be placed [and assessed!] inside or outside of. Like Ray McDermott’s critique of the traditional view of ‘context’, practice is neither “something into which someone is put, but an order of behavior of which one is a part” (McDermott, 1993, p. 290). Thus, a turn towards practice for EP also encompasses understanding how these educational practices, which educational psychologists are requested to approach, are continuously shaped by historical, structural, educational, social, and individual conditions. Importantly, educational psychologists and the work they do has been, throughout its relatively short history, part of this formation (this is theoretically elaborated in the next parts of the article). This means that the ‘practice’ that educational psychologists are requested to turn towards and thereby support is highly normative. The disconnection implies that EP practice risks being reduced to a series of *methodological* questions about how best to support educational practice, ensure more inclusive learning environments, or treat student anxiety etc. Without a situated understanding of the practice that educational psychologists are meant to advise, there is a significant risk of overlooking the more normative dimensions of practice (Szulevicz, 2021). An orientation towards practice is inherently an empty signifier – without specifying *which* practices to support, EP practice risks supporting practices that cannot be professionally justified.

*Second*, understanding the current idealized practice turn of EP practice as historically situated: it is neither just a ‘new’ discussion nor a ‘random’ discussion – it is connected to the historical struggles within the establishment and development of the field of EP and EP practice. The story of EP practice is deeply embedded in the modern prevalence of mass education in the western world, and therefore the requested turn towards practice needs to be understood as *situated* in modern institutionalized education.

*Third*, an idealized ‘turn towards practice’ is not new in the history of EP practice in and beyond Denmark. Since the late 1980s, the field of EP practice has changed (or more precisely, discussed the importance of changing its practice) from a primarily test-centered and biomedical school psychology towards a consultative framework highlighting the contextual, situated, relational nature of conflicts of children of concern (Anderman & Anderman, 2000; Bredo, 1994; Conoley & Gutkin, 1995; Conoley et al., 2020; Farrell, 2009; Szulevicz, 2025; Szulevicz & Tanggaard, 2017; Turner & Nolen, 2015). The current discussed practice turn could be seen as a new version of the same ideal: an increased focus on situating EP interventions closer to the ecologies, niches, and practices in which children and professionals engage (Kirkegaard Jensen & Szulevicz, 2024). This proclaimed development has been a heavily discussed ideal over the last 40 years of EP practice research. However, contemporary EP assessment practices are still dominated by psychometric measures and interventions focused on the child’s cognitive, emotional, social (dis)abilities. In 1995, Conoley and Gutkin – two prominent American researchers within the field of School psychology – asked *‘Why Didn’t – Why Doesn’t – School Psychology Realize Its Promise?’*. Furthermore, in 2020, they revisited this question: *‘How Is School Psychology Doing: Why Hasn’t School Psychology Realized Its Promise?* (Conoley et al., 2020). This paradox – over 40 years of advocating for a shift towards understanding and meeting children within their relational and contextual environments and everyday practices, now framed as a ‘practice turn’ – contrasts sharply the ongoing predominance of methodological concerns and isolated problem-focused practices in educational psychology. This situation calls for critical reflection on the historical role and function of EP practice and its embeddedness within the practice of institutionalized education: Why has this movement ‘towards practice’ been historically so difficult to achieve in EP practice?

The next part of the article is not a full-blown historical analysis of these questions, but an attempt to *situate* the current discussion of the requested practice turn in EP practice within selected, important milestones in the history of institutionalized education. When telling the story of EP and EP practice, it is common to tell it through the theories and conceptual developments of the dominant voices of education e.g. Stanley Hall, Theodore Thorndike, William James, John Dewey, Jean Piaget, Cyril Byrt, Godfrey Thomson, and so forth. Then the story of EP practice becomes a story of theoretical development, and the history of EP and EP practice is thus primarily understood through theoretical concepts and methodological tools. However, as described by Wright & Buchanan (2020, p. 499), “educational psychology did not simply emerge from the work of prominent researchers who in more hagiographical, and often gendered, accounts are termed founding fathers.” EP practice is not ‘something in itself’ – it is situated in the establishment and ongoing development of Western institutionalized education. To understand the current idealized ‘turn towards practice’ in EP practice in both a Danish, but also broader international context, the next part of the article attempts to situate EP practice within selected excerpts in the history of institutionalized education; the first focus is the birth of mass education in the late nineteenth and early twentieth centuries, the second focus is the period from 1980s and onwards focusing on the Western societal developments from industrial nations to knowledge societies and the function of education and educational psychologists within these changes.

## The Birth of Mass Education and How Educational Psychologists were Assigned a Special Role

The scientific field of EP emerged during the late nineteenth to early twentieth century a period when psychology transitioned from being a subfield of philosophy to becoming a specialized field of its own (Charles, 1976, 1987). Yet, the birth of EP as a distinct discipline of psychology must be understood in the context of the broader developments characterizing modern society (Nielsen & Tanggaard, 2018). As Boli et al. (1985, p. 145) noted, the profession of EP is linked to the prevalence of mass education as “a striking feature of the modern world”. During this period, the field of education was characterized by progressive ideals and social reforms in the Western world, and mass education introduced a new dilemma that led to the birth of EP practice:

In the context of the rise of mass schooling, selection was a key issue – at both ends of the ability spectrum. Godfrey Thomson, a contemporary of Burt’s, noted that psychologists were charged with the responsibility of “how with most justice to select eleven-year-old children in the primary schools for the privilege of free secondary school education” (Thomson, cited in Wooldridge, 2006, p. 70). Psychologists were also required to differentiate between those merely “intellectually dull and backward” from “mentally defective” children (London County Council [1911], cited in Wooldridge, 2006, p. 82) (Wright & Buchanan 2020, p. 505).

With the advent of mass education and the new sorting issue, the profession of EP practice was born. As Wright & Buchanan (2020, p. 496) describe, EP became a specialized area with “increasingly formalized applications to schooling”. In the Danish context, it was not new for ‘experts’ or other professional perspectives ‘from the outside’ to be part of the educational system. Since the Reformation, the church had supervised schools and teachers, a practice that continued into the first half of the nineteenth century (Appel & Fink-Jensen, 2013). However, from the mid-1800s, pedagogical attention was directed towards a new and growing (especially due to mass schooling) group of students – the ‘mentally and intellectually handicapped’ – and with this shift, new medical ‘experts’ entered the schools. The combination of increasing student numbers in school classes, especially in urban schools, increased demands on the school’s academic content (see the School Laws of 1814 in Denmark), and a new pedagogical interest in children’s diversities, behavior patterns, and abilities,^4^ led to a desire for a more ‘systematic classification’ of students. This period, influenced by social Darwinist thinking (see, e.g. the work of Ellen Key) focused particularly on so-called ‘abnormal’ children:

The abnormal could have both physical and psychological causes, and ‘abnormal children’ could thus be lame, blind and deaf, deeply mentally handicapped, mildly retarded, or for other reasons have difficulty keeping up with the teaching (Gjerløff & Jacobsen, 2014, p. 295).

During this period, a distinction was made between the ‘normal school’ for the majority of children and the ‘abnormal school’, which included various types of schools catering to children’s different needs. And precisely this division and the associated pedagogical work “required definitions of the various forms of abnormality, and especially within mental disabilities, the boundaries were fluid” (Gjerløff & Jacobsen, 2014, p. 295). Initially directed by medical science and the emergent scientific field of psychiatry, educational psychologists entered schools in the early twentieth century to develop and manage this classification and sorting work.

In 1934,^5^ the predecessor to EP, the first ‘school psychological office’ in Denmark, was established (Køppe, 1983). The background for its establishment began 20 years earlier. In the mid-1910s, a group of teachers and academics, united by a common interest in school development and a strong belief that psychology could contribute to this development (see also Wright & Buchanan 2020 for parallels in the broader history of Western educational psychology). This group of teachers and academics united in 1914 in the Association for Experimental Pedagogy with the aim of “initiating independent studies of school children” (Køppe, 1983, p. 115).

During this early modern period and until shortly after World War II, the Danish school psychological profession was established (Gjerløff et al. 2014). Initially, practical questions about organizing education for students with learning challenges were the focus of school psychology. However, with increasing attention to and interest in the concept of intelligence and intelligence testing both domestically and abroad, the primary task of school psychologists became to delineate what was considered normal and thus desirable in education (Nielsen, 2008; see also e.g. Charles, 1976; Wooldridge, 2006 in relation to its dominance in the broader field of educational psychology). In 1924, the Committee for School Psychological Studies was established in Denmark to work on intelligence and achievement tests in schools. Based on how children in different school classes worked with arithmetic, reading, and writing, the committee constructed a series of measures for what could be considered intelligence, with the standardized Binet-Simon intelligence test playing a central role (Køppe, 1983, see parallels in US and UK in e.g. Fagan, 2003; Leadbetter & Arnold, 2013). For example, in 1926, *Vor Ungdom*,^6^ a well-regarded educational journal of the time, wrote that “recently, attention has been drawn to the great societal significance that experimental psychology can have” (Gjerløff et al., 2014, p. 244). According to the journal, this type of psychological research could illustrate how teaching can be best organized by considering the individual child’s developmental stage.

During the initial period, the primary task and function of school psychology were to assess whether children were following the so-called ‘normal’ development. A significant societal backdrop for this sorting was the increasing and more specialized demands on the workforce during this period (Nielsen & Tanggaard, 2018; Dessent, 2022[1978]). As the Danish psychologist Simo Køppe (1983, p. 117) noted, one way to meet the need for more specialized labor was to establish a “system for channeling out the ‘social losers’, both because they hinder education and thus spoil efficiency, but also because the problems require special treatment”. Various special schools and special classes were established in Denmark during this period to segregate this group of students from the general education system to a special offer (see parallels in EP practice in many Western countries, Wright & Buchanan, 2020). In 1949, 15 years after the appointment of the first school psychologist, the ‘school psychological examination’ became mandatory in Denmark for a child to be referred for special education. By 1955, the Ministry of Education emphasized that school psychologists should conduct “intelligence measurement of children in school referred to him” (Bendixen, 2009, p. 204). Køppe (1983) notes that the school psychologists were tasked with measuring IQ, but it quickly became clear that solving these problems required psychologists to also formulate what reasonable treatment would entail and communicate these ‘educational principles.’ School psychologists were thus to test the student and based on that, suggest an appropriate intervention for the student’s continued school participation. Later in the article, we revisit the role of intelligence testing in the sorting tasks that EP practice was originally designed for. First we jump to the second period of interest, the period from 1980s and onwards.

## Education Within Knowledge Societies and New, Conflicting Tasks for Educational Psychologists^7^

The last 40 years of educational history takes place in a rapidly changing political reality. As described by Coninck-Smith, Rasmussen and Wyff (2015) in a very precise and concise narrative:

Denmark became a member of the EC in 1973 and later the EU. The Berlin Wall fell in 1989, and with the collapse of the Soviet Union two years later, the Cold War ended. The terrorist attack on the World Trade Center in New York in 2001 marked the beginning of an international fight against terrorism (…). Climate and sustainability came onto the political agenda, and successive governments from the right and left in the Danish Folketing (Parliament) began modernizing the welfare state from the 1980s (...). During the same decades, Denmark transformed from an industrialized nation-state to a globalized knowledge society, and competitiveness became a theme that increasingly influenced political debate in general, and the school debate in particular.

From the 2000s onwards, competitiveness became the normative framework of institutionalized education making performance testing and assessment key issues in educational practice. However, parallel to these frameworks, another narrative emerged in educational policy and practice. The educational practices of the 1990s and 2000s were influenced by migration from rural areas of Denmark to cities, as well as large migration flows from countries outside the EU to European, including Denmark. These changes altered the composition of children in the Danish educational system. In relation to these changes, the late 1990s saw the rise of discussions of ‘diversity’ including national and cultural identity (with globalization, the question of national identity also (re)emerged, see e.g. Buchardt, 2016; Gilliam, 2009). There was also a growing focus on ‘inclusion’ of diverse groups of children and youngsters in education, considering factors such as gender, ethnicity an. disability. The UN Convention on the Rights of the Child from 1989 and the Salamanca Statement of 1994 exemplify this shift in the understanding of the student or child in education. This period marked an important change in the conception of childhood (James & Prout, 2015[1990]), with children becoming subjects of rights and institutionalized education being understood as an important societal practice where children should learn about and experience these rights. Throughout the 2000s, theories related to these new views on children, such as ‘multiple intelligences’ (Gardner, 1983) ‘individual learning styles’ (Dunn & Dunn, 1999), ‘visible learning’ (Hattie, 2008) became popular. And as put by Coninck-Smith, Rasmussen and Wyff (2015, p. 17), it was “difficult to put the turn-of-the-century child into a formula; children rather appeared as unique individuals in their own right.”

Two dominant, and often conflicting normative, frameworks of institutionalized education have shaped the field from the 1980s to the present: competition and performance on one hand, and diversity and inclusion on the other. They share a focus on the *individual* child – they could be said to unite in a postmodern agreement to place the individual at the analytical center (yet, maybe for different reasons). However, they also differ significantly. The framework of competition and performance assessment does not have a purpose description, focusing primarily on how to succeed in competing rather than what to compete in or whether competition is necessary. Thus, this framework primarily becomes a question of methods. The framework of diversity and inclusion, on the other hand, has a normative, humanistic ideal and agenda: despite differences and inequities, children have the right to take part in and participate in educational practices, both concretely, but also emotionally to feel included in educational practices. The student ideal today thus balances between these two normative frameworks.

The developments within the field of EP practice over the last 40 years are embedded in these significant changes. However, this context is often overlooked when writing the history of EP practice. The story of EP practice during these years is frequently told as a story of a beginning test-critique and a movement towards consultation^8^ ( as if this critique of testing and ideals of consultation emerged solely from theoretical movements *alone* Kirkegaard Jensen & Szulevicz, 2024). This article aims to challenge the tendency to write this isolated story of EP practice. To understand why the practice turn requested of EP practice over the last 40 years has not occurred as expected, we need to explore what this practice entails and whether educational psychologists are truly distant from it, as described Looking into the developments within the profession of EP practice, from the turn of the millennium onwards, the consultative approach, where psychologists shift from an individual-oriented expert role to a more facilitating consultant position, has become a significant ideal. The beforementioned global advocacy for inclusive education, significantly propelled by the Salamanca Statement of 1994, redefined the role of educational psychologists, positioning them as consultants in fostering inclusive learning environments within schools (Farrell, 2004; Szulevicz & Tanggaard, 2015). However, despite widespread intentions and educational policy pressure for a more consultative practice, the psychometric basis continued to play a prominent role in EP practice in Denmark and beyond – and it still does (see e.g. Benson et al., 2019; Wilson & Reschly, 1996; Woods & Farrell, 2006).

The combination of consultation and testing became known as educational psychologists’ dual mandate (Moen et al., 2018) in EP practice, where the educational psychologists assess whether an intervention should be directed at the individual child or the child’s context. The coexistence of test-based and consultative practices has sparked ongoing debate, highlighting fundamental epistemological differences between the psychometric tradition and the contextual, relational understanding of consultation practice. This dual mandate reflects the two narratives of postmodern education – competition and inclusion. Educational psychologists’ role is at one and the same time to 1) assess a student’s performance-abilities in order to *sort* and *segregate*), and 2) to ensure that *every* child or student has the right to participate in the educational competition (that is, to support the pedagogical environment in relation to inclusive and legal rights to *stay* in the ‘educational competition’).

## Transformations and Relations Between the Two Periods

Looking across the two historical excerpts above, the ongoing development of the intelligence test stands as a central pillar in the development of educational psychologists’ function and professionalism both in Denmark and internationally (see e.g., Brown, 1992; Kaufmann, 2000; Nielsen, 2008; Ydesen, 2011; Ørskov & Ydesen, 2018; Wooldridge, 2006). The psychometric legacy and professionalism associated with conducting studies of “what is normal when” (Køppe, 1983, p. 117) are crucial to understanding the historical and current discussions about educational psychologists’ function and professionalism, including the desired turn towards practice. The psychometric legacy is not just a historically founded theoretical understanding with which school psychology or educational psychology practice as a discipline was born – an understanding that “[e]ven strong proponents of present-day educational testing concede (…) has a “dirty history” (Fletcher & Hattie, 2011, in Wright & Buchanan, 2020, p. 504). But it is more than just a ‘dirty history’. EP practice is historically situated in institutionalized education and has therefore become a *habitual practice* closely connected to educational psychologists both professionally and identity-wise. The question of sorting is still a dominant pattern in educational policy and practice. It has evolved with new ‘names and cultural forms such as the UN Convention on the Rights of the Child (e.g. Article 23 and 28) and the Salamanca Statement of 1994 changed how sorting, segregation and participation were *articulated* (Hall, 2019[1980], 2019[1986]) from the 1980s and onwards. Consequently, the description of educational psychologists’ function changed from primarily being understood as”test-and-place assessment roles” (Merrell, Ervin & Gimbel, 2012, p. 14) towards a new articulation as ‘agents of inclusions’ (Farrell, 2004).

However, these changing social formations did not fundamentally change the sorting issue of modern institutionalized education. Despite ‘inclusion’ becoming a dominant ideal in formal educational practices since the 1990s, educational practice and policy remain focused on comparing students’ behaviors and abilities, thus maintaining the sorting task of educational psychologists. With inclusion, it just added another – and conflicting – one. Thus, a turn towards practice in contemporary EP practice is also a turn towards a normative discussion of this sorting task of institutional education that educational psychologists were historically invited to take care of – and a job that institutionalized education is still inviting educational psychologists to take care of. This turn towards practice must include a discussion of the types of practices educational psychologists are currently supporting and *should* support, addressing the normative question of purpose in education (see e.g. Biesta, 2009, 2020; Martin & McLellan 2013).

The political, social, and cultural changes in the twentieth century are not a ‘distant backdrop’ (Coninck-Smith, Rasmussen & Wyff, 2015, p. 20) for the development of institutionalized education, but *an integral part of* this development. EP practice is deeply situated within these developments. Hence, the goals of the (educational) process (from religious education, character formation, democratic education, personal development, cultural identity, competitiveness etc.) are linked to historically changing societal practices and ideals about humans and their position and function in society. Educational psychology and the practice field of EP are embedded in this normative, changing practice and therefore it influences the type of ‘psychological assessments’ we make about appropriate, healthy, deviant, incorrect, sick, etc., behavior of children within society’s educational practices. Thus, the over 40-year-old dream about a turn towards practice, understood as a shift towards understandings of problems as situated in practice and contexts may not be unattainable due to wrong theories within EP practice. Instead, it may be challenging because educational psychologists’ task is not isolated but situated within normative educational practices.

In the last part of the article, a *situated psychology* framework of EP practice is presented to discuss the current practice turn in close relation to the historical struggles characterizing the field of EP practice.

## A Situated Psychology Framework for Understanding the Ideal of a Turn Towards Practice

In the current landscape, with increased attention on EP practice due to rising costs in special education and more children receiving psychiatric diagnoses, there are also higher expectations and demands on EP practice. One of these demands, as described in this article, is that EP practice should be situated closer to the everyday educational environment shared by students and teachers in schools. However, as the historical overview above illustrates, the relationship between EP practice and educational and school policy development has often been one where EP practice is reduced to a methodological concern about how best to support desired school development. This article argues that there is a need for greater historical and theoretical awareness of EP practice, so that it can more effectively address the normative questions that will always be part of the profession. Ultimately, this would also enable a more qualified discussion about the purpose of EP practice and connected to this, how then to understand and qualify the psychological knowledge that characterizes the specific psychological discipline of educational psychology in practice.

In the following, we will argue that the practice turn in EP practice can be qualified by theoretically incorporating it into a situated psychological framework. According to the introductory article of this journal, a situated psychological perspective posits that the social, material, and historical context is more than just the physical framework within which human behavior unfolds. The relationship between people and the social, historical context is understood as a prerequisite for understanding ‘the psychological’. As already mentioned, there are many different approaches to theorizing this relationship. Uffe Juul Jensen (2019) has described how, for example, the concept of practice since Plato and Aristotle and throughout the history of philosophy has given rise to lively discussions about the relationship between the individual and the state, as well as the organization and development of public institutions. Within the practice-philosophical tradition, Bernstein (1971), for example, writes, based on Marx’s concept of *praxis*, about practice as the concept for the dialectical understanding of humans as historically and socially produced – “[praxis] provides the perspective for grasping Marx’s conception of man as ‘the ensemble of social relationships’” (Bernstein, 1971, p. 13). In Kvale’s (1964, p. 195) words, humans live “in a specific historical situation and in a specific social relationship with their fellow humans. Human activity is primarily practice activity.” Situated learning theory draws on this understanding of practice (Lave & Packer, 2008). In situated learning, learning is understood not only as an individual process but as a social practice deeply rooted in the social, cultural, and historically developed contexts and contexts in which people participate. Learning cannot be reduced to a cognitive activity that takes place in the head of the individual person. Instead, it is a social practice that develops through our participation in communities and interaction with others.

For Lave and Wenger (2003[1991]), all activity is therefore ‘situated’ – all human practices must, in other words, be viewed as ‘situated’. However, Lave and Wenger point out that there is a lack of real awareness of the meanings of ‘the situated’. According to Lave and Wenger, the concept of situatedness should not be reduced to thoughts and actions that take place in space and time. Likewise, is it a misunderstanding if some activities are perceived as situated while others are not. The situated learning theory’s understanding of situatedness implies that the situated refers both to activities that take place within a specific and concrete situated practice, while also understanding that these same activities occur within a common political, historical, and cultural meaning system. The situated thus refers here to the dialectic between the participating people and the historical and concrete contexts in which these people’s activities are continually shaped and importantly, through people’s participation practice is continuously (re)shape (Lave, 1988) – in Lave’s (2004, p. 47) words: “participating in practice with others becomes the fundamental project that subjects engage in”. ‘Practice’ thus encompasses a fundamental relationship to *something* and *someone* (MacIntyre, 1985). Practice therefore refers both to our common world – the contexts and arenas we move in (schools, daycare centers, leisure clubs, homes, workplaces, etc., and the inherent meaning in these contexts) – and at the same time to our movement in and across these contexts – that is, our participation and ongoing (re)production and understanding of our common world. MacIntyre (1985) elaborates on such an understanding of practice, stating that practice should be understood as:

any coherent and complex form of socially established cooperative human activity through which goods internal to that form of activity are realized in the course of trying to achieve those standards of excellence which are appropriate to, and partially definitive of, that form of activity (p. 187).

For MacIntyre (1985), for example, politics and religions are examples of ‘practices’ – that is, practices are understood as both concrete and material (they can be historically and socially delimited) and they contain a moral order. There is something ‘at stake’ in practice or put differently: there are different normative ideals and expectations associated with our participation in different types of practices. In line with this, Bernstein (1971, p. xiv) writes about this moral or normative aspect of practice – “[praxis] signifies the disciplines and activities predominant in man’s ethical and political life.” Thus, in the practice-philosophical tradition, practice is not an expression of the mundane, a-theoretical, or merely ‘a place’ we go to ‘look’ when we, for example, need to see children ‘in situ’. Practices, such as e.g. school or daycare practices, are historically and socially produced activities with inherent normative, political, ethical, and moral meanings – and they are produced and reproduced, and as such, practice is also dynamic and something that develops over time.

In this article, we have employed such practice theoretical conceptualizations of *practice* as a situated psychological lens to *investigate* the apparent need for a practice turn in contemporary EP practice, as analyzed through a historical overview of the field. Additionally, we can use this lens to *unfold* how a practice turn in EP practice could or should ideally manifest, bearing in mind that this desired ‘practice’ is not a fixed structure, but a socially produced ‘reality’ co-created through everyday participation (e.g. as educational psychologists working in school or daycare settings). Returning to the quote from EVA (2023) presented in the introduction of this article, which highlights a deficiency in EP practice:

What is said to be missing is a connection between the psychological theoretical knowledge and a practice-oriented understanding of the school context and the competence to translate theoretical insights into this *practice*. (EVA, 2023, p. 61, our italics).

This ‘practice’ referred to, is not at all a fixed structure; it is far more complex. The quote from EVA suggests a dichotomous distinction between practice and theory. However, within a situated understanding of practice, practice should not be misconstrued as ‘non-theoretically oriented’. Following Bernstein (1971, p. xi) there may be a need to distinguish between “high and low senses of practical”. In line with, for example, the understanding of practice within pragmatism, the Marxist understanding of practice is far more sophisticated in the sense of ‘*high* sense of practical’, Bernstein (1971, p. xiv) writes:

”*Praxis*” in this more restricted sense signifies the disciplines and activities predominant in man’s ethical and political life, these disciplines, which require knowledge and practical wisdom, can be contrasted with “*theoria*” [this refers to Aristotle’s distinction between theoria and praxis] because their end is not knowing or wisdom for its own sake, but doing – living well.

So, if we again turn our attention to the desired shift towards practice, we need to add an important part and question to the discussion of this requested practice turn: what kind of practice do we as educational psychologists *want to* approach? This question must not be misunderstood as a ‘privileged position’ where educational psychologists in isolation can or should dictate what they want to do and support. On the contrary, we as educational psychologists need to grapple with the fact that EP practice is deeply situated within the practice of institutionalized education, and therefore co-producers of nationalized educational practices. So, if we – educational psychologists as well as all the stakeholders who have an interest in EP practice – want a practice turn, then we need to dare start a *joint* conversation about the purpose of educational practices and which educational practices we want to cultivate and support.

A *situated psychological* perspective that draws on practice theoretical conceptualizations when discussing the historical and current request for a ‘practice turn’ of EP, is thus more than just adding a theory that addresses the importance of bringing concepts as ‘context’, ‘practice’, or ‘system’ into the analysis of ‘children’ or ‘problems’ in school or daycare settings. As formulated by Bernstein above, it does not concern ‘knowing or wisdom for its own sake’ but the *normative* question of ‘doing – living well’.

For the last 40 years, researchers within the field of EP practice have described the importance of a shift from analyzing ‘individual problems’ to analyzing ‘situated problems’. In many ways, the authors of this article thus share the same interest as has been evident within the field, however, our understanding of ‘situated problems’ differ. In the historical key article by Sheridan and Gutkin from 2000, they too emphasize an ‘ecological theory’ as an important ‘conceptual base for the future’ (p. 489):

we propose that ecological theory, which conceptualizes human behavior as a function of ongoing interactions between the characteristics of individuals and the multiple environments within which they function, holds the greatest potential as an effective school psychological service-delivery orientation. (…) Success will hinge largely upon our ability to “*give psychology away*” (Miller, 1969, p. 1071) to individuals (e.g., teachers, parents, community leaders) who are integral to children’s natural, everyday, and long-term environments. Our job is build ecological systems that can support children, youth and families, and, in the final analysis, function effectively without us.” (p. 489, our italics).

In their elaboration on *how* such ecological systems are build and *how* they look like in practice, they e.g. unfold that,

(…) teachers must have the requisite skills to *implement interventions correctly* or be willing to learn these skills via in service, direct instruction, or modeling. Empirically supported interventions are unlikely to be effective when conducted incorrectly. (…) *School psychologists must find ways to present effective interventions to teachers so they are congruent with their philosophy of education* (p. 491, our italics).

Furthermore, in relation to the ‘macro-system’, Sheridan and Gutkin (2000) describe how ecological interventions must.

include services related to direct and indirect intervention, consultation, prevention, children’s mental health, family-centered services, and system support. It is the professional and ethical *responsibility of school psychologists to ensure that the manner in which policies are translated into practice* have the greatest potential to result in the greatest amount of benefit for children. (p. 494, our italics)

Even though ‘ecology’, ‘context’, ‘system’, or ‘practice’ are keywords in the ‘ecological theoretical’ perspective presented by Sheridan and Gutkin (2000) (as well as being evident in many other key publications connected to the requested ‘paradigm shift of EP practice’ dominant in postmodern research within the field of EP and EP practice especially around the turn of the twenty-first century, see e.g. Anderman & Anderman, 2000; Apter & Conoley, 1984; Conoley & Gutkin, 1995; Goodenow, 1992; Solomon, 1995), a *situated psychology* framework adds an important layer (and indirectly a critique thereof) to the ‘ecological’ understanding presented by Sheridan and Gutkin: ecology within a *situated psychology* framework is not just understood as ‘adding’ context to the individual layer. In contrast, it is a genuine understanding of human beings as inseparable of the global and local practices they continually shape and are shaped by. Thus, society and the individual are not conceived as separate ‘factors’ merely influencing one another, but as mutually constituted entities. This means, that when Sheridan and Gutkin in the quotes above (based on ecological theory heavily inspired by Bronfenbrenner, 1977, 1978) suggest that EPs should focus on ‘ecologies’, ‘ecology’ refers to what Gulløv and Højlund (2003) describe as a ‘traditional view of context’.

In a traditional understanding, context appears as a series of concentric circles surrounding the object of study; that is, the child is at the center, followed by the family, peer relationships, the classroom, the school as an institution, and finally the more abstract yet still framing society.” (Gulløv & Højlund, 2003, p. 33).

In this view, an ecological theoretical perspective becomes one where educational psychologists must remember to take any system or ‘circle surrounding the object of the study’ into account when considering interventions. Thus, from such outlook, an ‘ecological’ perspective is primarily framed as a methodological endeavor: e.g. *how* to ‘teach’ teachers to doing the right things *in practice* (Sheridan & Gutkin, 2000, p. 491); *how* to ‘translate’ policy *to practice* (Sheridan & Gutkin, 2000, p. 494). And more broadly summarized, how to ‘give psychology away’ (Sheridan & Gutkin, 2000, p. 489), thus, how to *deliver* theory and methods to all the systems and ‘individuals who are integral to children’s natural, everyday practices’ (p. 489).

In a situated psychology framework of EP practice and the requested turn towards practice, conceptualizations of ‘practice’ or ‘context’ or ‘ecology’ are not first and foremost a question of methods. From a situated psychology perspective, educational psychologists are not invited to consult or assess within a ‘neutral’ room – thus their job must never be reduced to a solely question of methods related to the problem of *translating* psychological theory into practice. From a situated psychology perspective on EP practice – drawing on practice theoretical conceptualizations of Lave, Bernstein and MacIntyre – our theories of e.g. children facing difficulties in teaching settings or with peers, are shaped by our common *participation* in the world (Lave, 2009). Thus, interventions that facilitate sustainable transformation in educational practice presupposes that we as educational psychologists pay close attention to our current and historical practices and our participatory habits, not just to our concrete methodological interventions. In the absence of critical engagement with our own involvement in shaping and perpetuating contemporary educational practices, we as educational psychologists risk normalizing practices that are ethically or professionally questionable.

In conclusion, the critical question is thus what type of practice educational psychologists should support in the movement towards a more practice-oriented approach in the field. As mentioned several times throughout this article, the practice that educational psychologists are to advise on is not without challenges. The ideals of inclusion are under pressure, the concept of normality is narrowing, and an increasing number of children are not attending school – to mention just a few. For educational psychologists to support practice effectively, a fundamental theoretical and normative discussion is required. This discussion should consider the profession’s historical development and its connection to the school system, enabling a qualified reflection on how the EP profession can both counteract and simultaneously reproduce marginalization processes affecting children and young people.

For too many years, the EP profession has been preoccupied with developing approaches and methods to support inclusion, treat anxiety, or address other issues. However, without a fundamental (theoretical) understanding of what makes inclusion difficult or why students develop anxiety, there is a risk of engaging in unreflective symptom treatment for issues that should be addressed differently. Similar concerns can be raised regarding the demands for more practice-oriented EP counseling. If the practice-oriented approach is merely reduced to a contentless, atheoretical, and methodological matter, it becomes just another requirement for the profession to meet. Conversely, if it is accompanied by a theoretical and normative purpose-driven discussion, it may serve as a professional lever for the field.

## Data Availability

No datasets were generated or analysed during the current study.

## Footnotes

1 Danmarks Lærerforening (The Danish Union of Teachers).

2 Børne- og UngdomsPædagogernes Landsforbund (The National Federation of Child and Youth Educators/Pedagogies).

3 Kommunernes Landsforening (The National Association of Municipalities).

4 The *Child Study movement* in the US in the late 1800s was a dominant movement (e.g. Florell, 2018) as well as the intelligence test developed by Alfred Binet in France in 1905 got an enormous influence of the field (Nielsen, 2008).

5 In the article we focus on the field of EP practice in Denmark, however, the development in Denmark is closely connected to developments of the profession of EP in the Western world, especially UK and US. In UK the first educational psychologist was appointed in 1913 (Leadbetter & Arnold, 2013). In the US, Arnold Gesell was appointed in 1915 as the first to hold the title ‘school psychologist’ (Fagan, 1992, p. 240).

6 In English’Our Youth’.

7 In the analyses of’institutionalized education’ from 1980s onwards, we zoom in on the school institution. However, in this period the daycare institution becomes a very important new context for the work of educational psychologists. But due to both space and aims of this article, we only focus on the school, not because of daycare centers being of minor importance, but because it calls for a deeper analysis of the historical developments of daycare institutions and their societal function. Including the growing focus on ‘early prevention’ (in a Danish context, see e.g. Børne- & Undervisningsministeriet [Ministry of Children and Education], 2024; Hvidtfeldt & Stanek 2022; Kjær & Olesen, 2005).

8 In the American school psychology tradition for example, this was presented as a critique of the predominantly ‘medical model’, which focused on individual assessments and diagnostics (e.g., Conoley & Gutkin, 1995; D’Amato et al., 2011; Gutkin & Conoley, 1990). Around the turn of the millennium, this criticism culminated in a call for a paradigm shift in school psychology practice: “Our longstanding professional dilemmas are not the result of practitioners, trainers, and researchers somehow doing a poor job of what it is that they do. The problem is much more basic. We are doing the wrong things for the wrong reasons. […] Our role conceptualizations and professional behaviors must change qualitatively and fundamentally. Coping with the anomalies of service delivery for children, youth, and families will require school psychology to move from the medical model and its nearly exclusive child-centered focus toward an ecological and multilevel systems paradigm for school psychological services” (Sheridan & Gutkin, 2000, p. 488). The proposed paradigm shift drew theoretical inspiration from a wide range of theories, including ecological systems theory (Bronfenbrenner, 1977; Sheridan & Gutkin, 2000) and consultation theory (Shapiro, 2000), which, despite differences, shared a common orientation towards relational, contextual, and situated problem understandings. This shift can be linked to the ‘relational turn’ within the humanities and social sciences from the 1980s onwards (e.g., Dépelteau, 2008; Desmond, 2014; Emirbayer, 1997).
